# Comparison of three treatment methods for simple bone cyst in children

**DOI:** 10.1186/s12891-020-03933-8

**Published:** 2021-01-12

**Authors:** Ke-Xue Zhang, Wei Chai, Jia-Jia Zhao, Jun-Hao Deng, Zhan Peng, Ji-Ying Chen

**Affiliations:** 1 Medical School of Chinese PLA, Beijing, 100853 China; 2 Department of Orthopaedics, Chinese PLA General Hospital, Beijing, 100853 China; 3 Department of Anesthesiology, Shun Yi District Hospital, Beijing, 101300 China

**Keywords:** Child, Bone cyst, Elastic stable intramedullary nail, Autologous bone marrow

## Abstract

**Background:**

The unicameral bone cyst (UBC) is a kind of benign tumor whose clinical treatments and efficacy are controversial. The purpose of this study was to evaluate the efficacy of the elastic stable intramedullary nail (ESIN), the injection of autologous bone marrow (ABM), and the combination of ESIN and ABM in the treatment of bone cyst in children.

**Methods:**

Eighty-three cases with simple bone cyst were analyzed retrospectively. Twenty-eight cases were treated with ABM. Twenty-eight cases were treated with ESIN. Twenty-seven cases were treated with ABM and ESIN. All cases were diagnosed through X-ray, CT, or MRI scans. For the suspicious ones, the pathological biopsy was performed for an accurate diagnosis. X-ray examinations were carried out for the postoperative follow-up. Capanna criteria for bone cyst was used for postoperative evaluation of three methods.

**Results:**

All cases accomplished the follow-up. The effective rate of the ABM + ESIN group was significantly higher than that of the ABM group (*P* < 0.05), and the cure rates of the ESIN group and the ABM + ESIN group were higher than that of the ABM group (*P* < 0.05, respectively). The cure time in the ESIN group was lower than that of the other two groups (*P* < 0.05, respectively). The times for admission were 2.0 ± 0.0 in the ESIN group, 5.7 ± 1.9 in the ABM group, and 4.7 ± 2.4 in the ABM + ESIN group (*P* < 0.05 when compared with each other).

**Conclusions:**

The method of ABM combined with ESIN for children’s bone cyst has the highest effective rate and curative rate. For the individual method, ESIN has a higher effective rate and curative rate than that of ABM. Meanwhile, it has the fewest time of hospitalization.

## Background

The unicameral bone cyst (UBC) is a kind of benign tumor that usually occurs in the epiphysis of the long diaphysis [1–3]. The etiology and pathogenesis of UBC in children have not been identified [3]. There are many treatments in clinical practice for bone cysts, such as autograft or allograft bone grafting after lesion curettage [3–5], local injection of autogenous bone marrow (ABM) [6, 7] or methylprednisolone [8], and implantation of the elastic stable intramedullary nail (ESIN) [9–11] et al. Among them, local injection of ABM and implantation of ESIN were used widely. However, the comparison of clinical efficacy among ABM, ESIN, and ABM combined with ESIN is rarely reported. In this study, we tried to analyze the three methods in a total of 83 cases retrospectively to disclose and compare their efficacy. Twenty-eight cases were treated with ABM, 28 cases were treated with ESIN, and 27 cases were treated with ABM combined with ESIN. All patients were evaluated by preoperative and postoperative X-ray examination. The Capanna evaluation criteria [12] was used to compare the clinical efficacy.

## Methods

### Ethical approval

The study was approved by the ethical committee of the Chinese PLA General Hospital. Written informed consent for this study was obtained from all children’s parents.

### Inclusion and exclusion criteria

#### Inclusion criteria

(1) Age was under 14 years old. (2) Diagnosis was ascertained by X-ray, CT, or MRI images. For the suspicious ones, the pathological biopsy was performed. (3) No treatment was performed before admission.

#### Exclusion criteria

(1) Lesions were complicated with other neoplasms. (2) Pathological fractures occurred in the processes of internal and external fixations.

### Criteria for diagnosis

In X-ray plains, the simple cyst presents with a round or oval low-density area with mild plumping. Its long axis is mostly parallel to the shaft. Bone ridge separation is visible inside of the bone cyst. The boundary of the cyst is clear and sharp, mostly with the thin-wall sclerotic edge. Bone debris collapse sign can be found when pathological fracture happens [1, 13].

### Patients data

Eighty-three children with bone cysts were retrospectively studied. They were admitted to the Department of Pediatric Surgery of Chinese PLA general hospital from January 2010 to December 2016. Twenty-eight cases were treated with ABM. Twenty-eight cases were treated with ESIN. Twenty-seven cases were treated with ABM combined with ESIN. The general data of patients were shown in Table 1. There was no statistical significance in age, gender constitution, weight, and height among the three groups.

**Table 1:** General clinical data in 83 cases among three groups

Group	Cases	Gender		Age	Weight	Height	Follow-up time	Location of cyst				
		Male	Female	(x̄± s,year)	(x̄ ± s,kg)	(x̄ ± s,cm)	(x̄ ± s,month)	proximal humerus	proximal femur	proximal tibia	femoral shaft	distal femur
ABM	28	16	12	7.7 ± 2.0	17.6 ± 4.0	121.9 ± 7.3	32.4 ± 9.2	10	8	6	4	0
ESIN	28	18	10	7.5 ± 3.1	18.1 ± 3.0	120.8 ± 6.1	32.1 ± 3.2	11	7	5	4	1
ABM + ESIN	27	17	10	7.7 ± 2.3	18.3 ± 4.0	122.7 ± 7.0	31.7 ± 6.4	10	6	5	4	2
F	–	0.3		0.1	2.2	0.5	0.7	2.5				
P	–	0.8		0.9	0.3	0.5	0.7	1.0				

### Surgical procedures

**Fig. 1:**
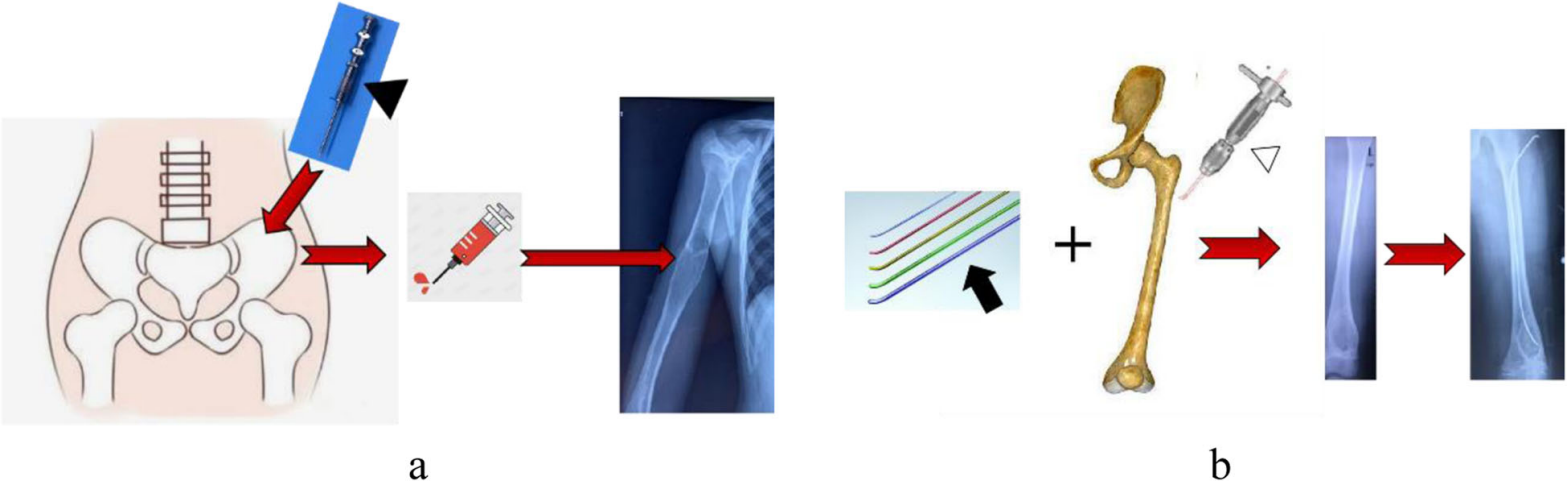
The diagrams of autologous bone marrow injection (1**a**) and elastic stable intramedullary nail implantation (1**b**). 1**a** The bone marrow blood was extracted from the ilium using a bone penetration needle (short solid arrowhead) and injected into the femoral cyst with the guidance of the X-ray fluoroscopy. 1**b** The holding device (hollow arrowhead) was used to implant the elastic intramedullary needle (long solid arrow) into the femur with the guidance of the X-ray fluoroscopy

ABM Injection [14]: After general anesthesia with endotracheal intubation, the operative area were sterilized and laid with sterile sheets. Under the guidance of X-ray fluoroscopy, a core-inside trocar was punctured to the bone surface adjacent to the cyst. Then it was pierced into the center of the cyst slowly. When the inside core of the trocar was withdrawn, the liquid of the bone cyst outflowed. Next, ABM extracted from the Iliac crest was injected into the bone cyst cavity slowly. After the completion of the ABM injection, the trocar was removed. The puncture points were covered with sterile dressings. The diagram of ABM injection was shown in Fig. 1a.

ESIN Implantation [14]: After general anesthesia with endotracheal intubation, the operative area were sterilized and laid with sterile sheets. Under the guidance of X-ray fluoroscopy, a 1 cm incision was made at the epiphysis of the long diaphysis away from the bone cyst. A hemostatic forceps was used to dissect the subcutaneous tissue to the bone cortex. An electric drill was used to drill a hole in the bone cortex. The ESIN, pre-bent into a “C” shape, was slowly inserted into the medullary cavity along the drill hole with the guidance of X-ray fluoroscopy. When the ESIN was properly implanted, the outer portion of the nail was bent and cut for complete subcutaneous embedding. Then the incision was sutured and wrapped with sterile gauze. The diagram of ESIN implantation was shown in Fig. 1b.

ESIN+ABM method: After one to three times of ABM injection with the interval of three-month in each time, the ESIN was implanted.

All cases were given postoperative antibiotics of cefuroxime sodium for 2 days. The affected limbs were fixed with plaster for 6 to 8 weeks. All cases were encouraged to early exercises after surgery. For cyst cases of lower extremities, they were encouraged to weight-free exercise in bed in the early period of recovery in order to avoid pathological fracture.

### Follow-up and outcome appraisal indicators

The therapeutic effect of treatment was evaluated by the criteria of Capanna [12] for the bone cyst. All children received preoperative X-ray, CT, or MRI examinations. The pathological biopsy was carried out if necessary. All patients were followed up with X-ray examinations. Preoperative and Postoperative images evaluation were performed for all the patients. In the process of Capanna evaluation, two experienced clinicians conducted the double-blind evaluation. When there was a difference in the scores of the same patient, the third specialist participated. The evaluation criteria of Capanna [12] for bone cyst: (1) Complete cure: The cyst cavity is completely filled with new bone. No residual lesion is observed. (2) Residual Cure: Lesion area is mostly replaced by newly growing bone tissue. The mixture of newly growing bone with the surrounding cyst wall bone can be seen. The cyst wall of the cortex sclerosis thickens. Small transparent areas present in the original cyst site. (3) Recurrence: In the early stage of the treatment, a good effect can be observed. Subsequently, transparent areas in the original cyst cavity emerged again. Bone cortex around the cyst becomes thinner. (4) No response: X-ray shows no favorable changes and healing tendency.

Effective cases included complete cure cases and residual cure cases. The effective rate was calculated by the proportion of effective cases to the total number of the cases being treated.

### Statistical analysis

SPSS 20.0 statistical analysis software was used for statistical analysis. One-way ANOVA test was used in the analysis data of admission times, age, and height. Kruskal-Wallis rank-sum test was used in the analysis of weight, follow-up duration, and therapeutic times for cure ones. Chi-square test was used in the analysis of gender composition, effective rate, cure rate, and the site of cyst. *P* < 0.05 was considered statistical significance.

## Results

### The comparison of three methods in the treatment of bone cyst in children

**Fig. 2:**
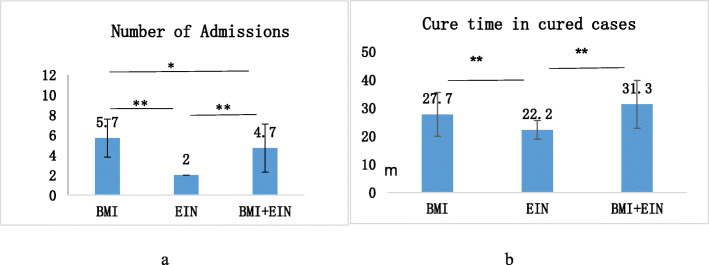
The comparison of the hospitalization time and the cure period in the three groups. 2**a** The number of admissions. 2**b** Cure time in the cured cases. One-way ANOVA test was used. *P < 0.05, ** *P* < 0.01 indicates the statistical significance

All the cases accomplished the follow-up. The follow-up times were 32.1 ± 3.2 months in the ESIN group, 32.4 ± 9.2 months in the ABM group and 31.7 ± 6.3 months in the ABM + ESIN group (Table 1). In the ESIN group, 25 cases were healed (23 cases cure, two cases residual cure), two cases recurred, one case did not respond to the treatment. In the ABM group, 18 cases were healed (13 cure, five residual cure), eight cases recurred, and two did not respond to the treatment. In the ABM + ESIN group, 26 cases were cured and one case residual was cured (Table 2). The effective rate of the ABM + ESIN group was significantly higher than that of the ABM group (*P* < 0.05), and the cure rates of the ESIN group and the ABM + ESIN group were significantly higher than that of the ABM group (*P* < 0.05, respectively). Among three groups who were completely cured (23 in the ESIN group, 13 in the ABM group and 26 in the ABM + ESIN group), the cure period was 22.2 ± 3.3 months in the ESIN, 27.7 ± 7.8 months in ABM, and 31.3 ± 8.5 months in the ABM + ESIN group. The cure period of the ESIN group was significantly lower than that of the other two groups (*P* < 0.05). The frequency of hospitalization was 5.7 ± 1.9 times in the ABM group, 2.0 ± 0.0 times in ESIN group, and 4.7 ± 2.4 times in ABM + ESIN group. Pairwise comparisons between the three groups were statistically significant (*P* < 0.05) (Fig. 2).

**Table 2:** The comparison of three methods in the treatment of bone cyst in children

Group	Case	Number of effective cases (effective rate)	Cured cases (cure rate)
ABM	28	18(64.3%)	13(46.4%)
ESIN	28	25(89.3%)	23(82.1%)^*^
ABM + ESIN	27	27(100%)^*^	26(96.3%)^*^

X^2^ test was used to compare the therapeutic effect among the three methods. Compared with ABM group, ^*^P < 0.05 indicates the statistical significance

### Case one

Figure 3

**Fig. 3:**
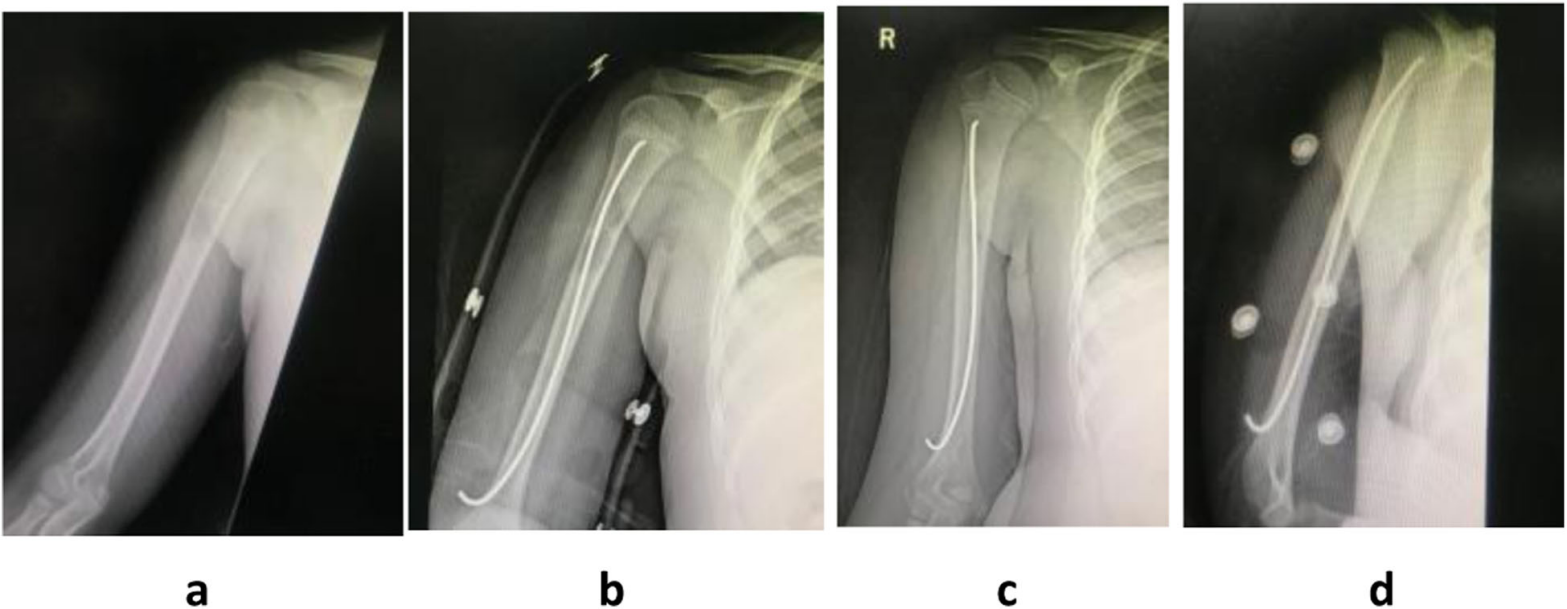
Male, eight years old, unicameral bone cyst localized in his right proximal humerus. 3**a** X-ray before the implantation of ESINs. 3**b** X-ray image after the implantation of ESINs. The ESINs runs through the cyst. 3**c** X-ray image at three months after the operation and there is new osteotylus in the cyst. 3**d** X-ray image at six months after the operation showed more osteotylus formed in the cyst. The bone cyst was healed

### Case two

Figure 4

**Fig. 4:**
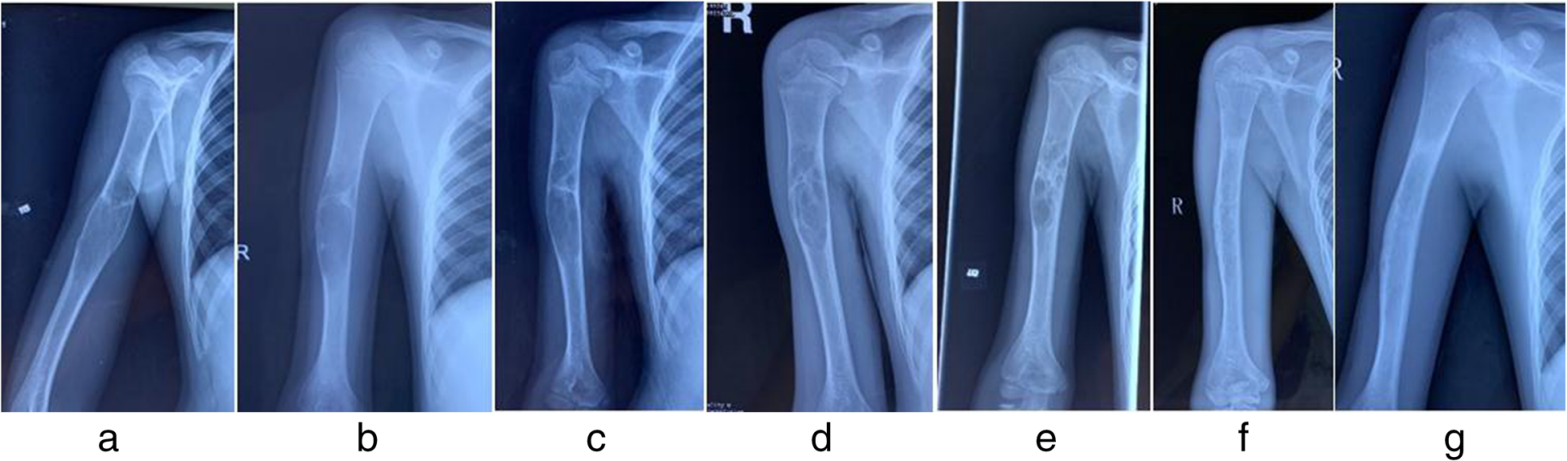
Male, nine years old, the bone cyst localized in his right humeral bone. 4**a** X-ray image before the first ABM injection therapy. 4**b** X-ray image prior to the second ABM treatment (3 months after the first ABM injection). 4**c** X-ray image before the third ABM treatment (6 months after the first ABM injection), the formation of the newly growing bone callus in the cyst can be seen. 4**d** X-ray image before the fourth ABM treatment (1 year after the first ABM injection), more callus bone could be seen. 4**e** X-ray image before the seventh ABM treatment (30 months after the 1st bone marrow blood injection), bone cyst is divided into multiple cystic spaces by the newly growing bone. 4**f** 42 months after the first ABM injection, the bone cyst was healed. 3 **g**: 56 months after the first ABM, the bone cyst was cured

### Case three

Figure 5

**Fig. 5:**
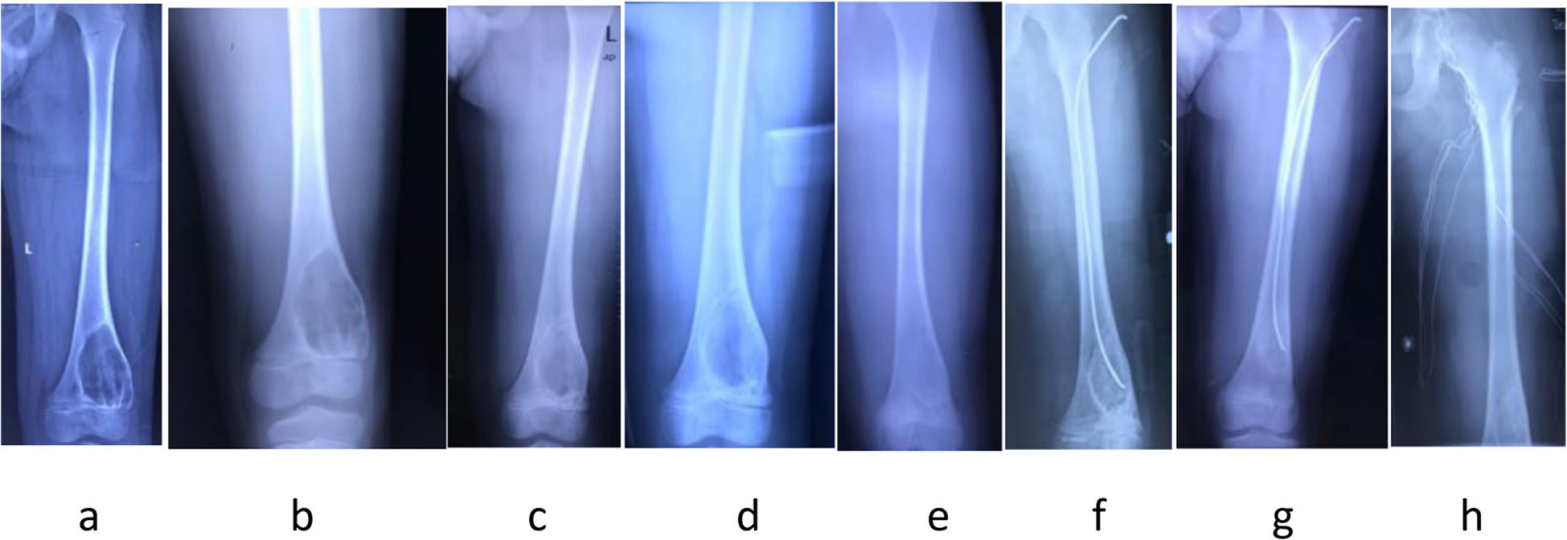
Male, eight years old, unicameral bone cyst localized in his right distal femur. 5**a**-5**b** The X-ray plains of preoperative and postoperative of ABM injection for the first time. 5**c**-5**d**: The second ABM injection was performed 5 months later. 5**e**-5**f** 5 months later, the ESIN was inserted. 5 **g**-5 **h**: 14 months later, the ESIN was removed and the bone lesion was cured

## Discussion

The bone cyst has been studied for more than 100 years, but its pathogenesis and etiology are still unknown. Chigira et al. [15] believed that the increase of intracapsular pressure caused by the stasis of the intraosseous vein resulted in the accumulation of a large amount of exudate in the epiphysis of the diaphysis and led to the localized osteonecrosis of the epiphysis, which eventually led to the formation of the bone cyst. Injection of ABM can provide growth factors and mesenchymal cells for the cystic cavity. Mesenchymal cells have a variety of differentiation potentials and can differentiate into osteogenic cells, then the newly growing bone tissue may be anticipated. This study showed that ESIN had the therapeutic advantage of a shorter curative period and hospitalization time when compared with that of ABM. ABM provides mesenchymal cells and related growth factors locally, but the pathological condition of cyst formation remains and leaves a high risk of recurrence and refracture [16]. The treatment principles of ESIN are as follows (1) Decompression and drainage of the cyst fluid. Drainage can reduce the pressure within the cyst and even remove the adverse factors that hinder the healing, such as PGE2, lysosomes, toxic free radicals, and interleukin-2. (2) ESIN provides support inside the bone marrow cavity. (3) ESIN promotes the growth of new bone around the intramedullary nail. Many scholars also believe that ESIN is the best treatment for patients with simple bone cyst. Roposch et al. [11] treated 32 patients with bone cyst with ESIN, with an average follow-up time of 105 months, and found that the cure rate was as high as 94%。De Sanctiset al [10]. treated 47 patients with bone cyst treated by ESIN, with an average follow-up time of 11 years, and found that the cure rate was as high as 100%.

Our study suggested that when compared with the previously reported methods of local curettage and bone grafting [12], ESIN had a higher cure rate and shorter cure period. For the local curettage and bone grafting method, although the lesion is removed ephemerally, the pathological condition causing the bone cyst formation persists. ABM has the advantages of simplicity and safety, less pain in a single surgery and wide source of bone marrow. Its disadvantages include poor long-term treatment effect, high recurrence rate, high cumulative treatment cost, the need for multiple times of surgeries, and cumulative pain of overall treatment. Hence, ESIN is more worthy of recommendation in the treatment of simple bone cyst in children.

It should be emphasized that there are some complications and risks associated with ESIN [17]. Most bone cysts occur at both ends of the long bone and close to the epiphyseal plate. In the case whose cyst is adjacent to or invades the epiphyseal periphery, improper implantations of the ESINs easily cause damage to epiphysis plate, which will result in a negative effect on the growth and development of the bone. Furthermore, when the cyst occurred in a thin bone. ESIN could easily penetrate outside of the bone cortex (such as case one), which will lead to the damage of surrounding blood vessels and nerves. ESIN tail can often lead to the irritant reaction of the subcutaneous tissue, which may cause local inflammatory hyperplasia and ulceration. For the avoidance of the epiphysis plate injury, if the implantation of ESIN becomes superficial, it will compromise the function of supporting and draining which will lead to unsatisfactory therapeutic effect. Hence, we propose the ABM method for this kind of patients in the early stage of therapy. When a new callus in the cyst presents, ESIN combined with ABM was recommended to reduce the following surgical manipulations. Our study showed that the combination of ABM and ESIN has the advantages of two methods, especially for the bone cysts adjacent to the epiphyseal plate. It has the characteristics of high efficiency and a high cure rate.

The limitation of this study was that it was a retrospective study with a short follow-up period. The average age of patients was around 7 years old in all 3 groups and the overall follow-up was 32 months (average) in all groups. The follow-up period needs to be extended in the future. Meanwhile, further mechanism study of the combination therapy of ABM and ESIN should be continued.

## Conclusion

ABM combined with ESIN has a definite efficacy for the treatment of children’s bone cyst with a high cure rate and controllable treatment process, especially for the cases of cysts adjacent to the epiphysis periphery or even evade the epiphysis plate. This method is worthy of clinical application. For the individual method, ESIN is better than that of ABM with less hospitalization time and a shorter cure period.

## Data Availability

Data associated with this study is retained at a central repository at the Department of Pediatric Surgery, Chinese PLA General Hospital. All imageology acquisitions were undertaken at the Department of Diagnostic Radiology, Chinese PLA General Hospital. The datasets used and analysed during the current study available from the corresponding author on reasonable request.

## Abbreviations

UBC: Unicameral bone cyst
ABM: Autogenous bone marrow
ESIN: Elastic stable intramedullary nail
CT: Computerized tomography
MRI: Magnetic Resonance Imaging
ANOVA: Analysis of variance
PLA: Chinese People’s Liberation Army
